# Men and COVID-19: A Biopsychosocial Approach to Understanding Sex Differences in Mortality and Recommendations for Practice and Policy Interventions

**DOI:** 10.5888/pcd17.200247

**Published:** 2020-07-16

**Authors:** Derek M. Griffith, Garima Sharma, Christopher S. Holliday, Okechuku K. Enyia, Matthew Valliere, Andrea R. Semlow, Elizabeth C. Stewart, Roger Scott Blumenthal

**Affiliations:** 1 Center for Research on Men’s Health, Vanderbilt University, Nashville, Tennessee; 2 Ciccarone Center for Prevention of Cardiovascular Disease, Division of Cardiology, Johns Hopkins University School of Medicine, Baltimore, Maryland; 3 Improving Health Outcomes, American Medical Association, Chicago, Illinois; 4 The George Washington University Milken Institute School of Public Health, Washington, DC; 5 CareSouth, Baton Rouge, Louisiana; 6 Meharry Medical College, Nashville, Tennessee

## Abstract

Data suggest that more men than women are dying of coronavirus disease 2019 (COVID-19) worldwide, but it is unclear why. A biopsychosocial approach is critical for understanding the disproportionate death rate among men. Biological, psychological, behavioral, and social factors may put men at disproportionate risk of death. We propose a stepwise approach to clinical, public health, and policy interventions to reduce COVID-19–associated morbidity and mortality among men. We also review what health professionals and policy makers can do, and are doing, to address the unique COVID-19–associated needs of men.

**Summary**

**What is already known about this topic?**

Data suggest that more men than women are dying of coronavirus disease 2019 (COVID-19) worldwide, but it is unclear why.

**What is added by this report?**

We describe an approach that considers biological and psychosocial factors that affect men’s health and how these factors may intersect. Clinical, public health, community, and policy examples illustrate what can be done, and is being done, to address men’s COVID-19–associated mortality risk. Our approach highlights the importance of examining COVID-19–associated mortality risk from a men’s health perspective rather than one that focuses solely on sex differences.

**What are the implications for public health practice?**

We can seize this moment to reimagine and redesign our health care and public health systems to consider the many factors that influence men’s health.

## Introduction

The novel coronavirus disease 2019 (COVID-19) is shining a spotlight on the neglect of men’s health at local, state, national, and global levels (1). According to the largest body of publicly available sex-disaggregated data from global government sources, although no apparent sex differences exist in the number of confirmed cases, more men than women have died of COVID-19 in 41 of 47 countries (2), and the overall COVID-19 case-fatality ratio is approximately 2.4 times higher among men than among women (3,4). In the largest survey of 72,314 suspected or confirmed cases of COVID-19 in China (men, 63.8% of cases; women, 36.2% of cases), the case-fatality ratio was higher among men (2.8%) than among women (1.7%) (5). Another study from China, of critically ill patients, showed that men with comorbidities such as hypertension, cardiovascular disease, chronic kidney disease, and diabetes had the highest mortality (6) and US data showed similar patterns (4,7,8).

A report on 3,200 COVID-19–related deaths from Italy showed higher death rates among men than women across all age groups, with men accounting for more than 70% of deaths (3). A multinational health research database using the TriNetX Network showed that among 14,712 male and female patients with confirmed COVID-19, men were older, were more likely to be hospitalized, and had a higher prevalence of hypertension, diabetes, coronary heart disease, obstructive pulmonary disease, nicotine dependence, and heart failure. Men also had higher all-cause mortality than women (8.1% vs 4.6%) (9). Moreover, the cumulative probability of survival was significantly lower among men after adjusting for age, comorbidities, and use of angiotensin-converting enzyme inhibitors (ACEIs) or angiotensin receptor blockers (ARBs) (9).

In the United States, as of June 2020, 57% of deaths caused by COVID-19 have been men. With the exception of Massachusetts, all states in the United States have reported higher mortality among men (10). However, the United States has not been consistent in reporting sex-disaggregated data. In a recent analysis of 26 states, only half reported sex as a variable (10). Age is a significant risk factor for COVID-19 mortality, and a vast majority of the COVID-19 deaths in the United States has been among people older than 75; in addition, rates of preexisting health conditions (eg, hypertension, obesity, diabetes) exacerbate disparities in mortality by class, race, and sex/gender (8). Exploring the differences in COVID-19 morbidity and mortality across these sociodemographic strata are beyond the scope of this commentary, yet we recognize and note that race, ethnicity, sexual orientation, gender identity, and other factors are important and should call attention to particular populations during the COVID-19 pandemic.

In this commentary, we discuss factors that may put men at a disproportionate risk of dying of COVID-19. Although it can be useful to compare determinants of men’s health to those of women’s health, our approach helps to identify why, how, and under what conditions key determinants of health affect the health outcomes of men (11). This approach facilitates efforts to identify strategies to intervene and improve the health of men during this public health crisis and beyond (12). After we examine the determinants of men’s risk of dying of COVID-19, we describe what medical providers, public health professionals, and policy makers can do, and have been doing, to address the unique needs and risks among men.

The sex gap in COVID-19–associated mortality is not easily explained by any single biological or social factor (3). Recognizing the difference between sex and gender in health outcomes while discerning the influences one has on the other is important (13). Differences in sex are biological. These include differences in reproductive organs and their functions, sexual hormones, and the gene expression of chromosomes. Gender is the performance of socially constructed roles, behaviors, and attributes considered socially acceptable for men and women. Consequently, we use a biopsychosocial approach that considers biological and psychosocial factors that affect men’s health and how these factors may intersect (14).

## Factors Affecting COVID-19 Morbidity and Mortality Among Men

Although epidemiological data show a difference between men and women in the rates of mortality among those diagnosed with COVID-19, the mechanisms underlying sex differences in mortality are unclear (3,10,15). Because most health patterns are the result of a combination of biological, behavioral, and psychosocial factors, we must consider how sex-associated biological factors and gender-associated psychosocial and behavioral factors interact in determining health (14) and in explaining COVID-19–associated mortality (4,8,15). In this section, we first describe biological factors and then discuss psychological and behavioral factors associated with men’s higher risk of COVID-19–associated mortality.

### Biological factors

Men and women differ in both innate and adaptive immune responses, perhaps related in part to sex-specific inflammatory responses resulting from X-chromosomal inheritance. The X chromosome contains a high density of immune-related genes; therefore, women generally mount stronger innate and adaptive immune responses than men (3). This differential regulation of immune responses in men and women is contributed by sex chromosome genes and sex hormones, including estrogen, progesterone, and androgens. Sex-specific disease outcomes after viral infections are attributed to sex-dependent production of steroid hormones, different copy numbers of immune response X-linked genes, and the presence of disease susceptibility genes (3).

Severe acute respiratory syndrome coronavirus 2 (SARS‐CoV‐2) uses the SARS‐CoV receptor angiotensin-converting enzyme 2 (ACE2) for entry into the host cell (16). The S spike of the virus attaches to the cellular ACE2 receptor (coded by the ACE2 gene) located on the respiratory epithelial cells. The internalization of the virus is potentiated by the cellular protease TMPRSS2 (transmembrane protease, serine 2) in the host cell (17,18). The high burden of illness and high case-fatality ratio in patients with COVID-19 may be driven in part by the strong affinity of the virus for ACE2, leading to virus entry and multisystem illness in pulmonary, gut, renal, cardiac, and central nervous systems (16).

Men have higher plasma ACE2 levels than women do, and a recent study of patients with heart failure showed that plasma ACE2 concentrations were higher than normal in men and higher in men than in women, possibly reflecting higher tissue expression of the ACE2 receptor for SARS‐CoV infections (19). This could explain why men might be more susceptible to infection with, or the consequences of, SARS-CoV-2. Unravelling which cellular factors are used by SARS-CoV-2 for entry might provide insights into viral transmission and reveal therapeutic targets. Further investigation into the association of ACE2 enzyme activity in COVID-19 and its correlation with sex is ongoing. Although biological factors clearly help to explain the sex difference in COVID-19 mortality, psychosocial and behavioral factors also play a part.

### Psychosocial and behavioral factors

In addition to sex differences in immune responses, hormones, and genes, there are also psychological, social, and behavioral components that influence COVID-19 progression (1,15). Compared with women, men tend to engage in more high-risk behaviors that generate potential for contracting COVID-19 (1,4). Polls taken early in the first wave of COVID-19 cases in the United States show sex differences in the perceived severity of the pandemic (20). Another US study found that men have been more likely to downplay the severity of the virus’s potential to harm them (21), and fewer men than women have reported that they have been avoiding large public gatherings or avoiding close physical contact with others (21–23). In addition, compared with women in many countries, including the United States, men tend to have higher rates of behaviors that are linked with COVID-19 infection and mortality, including higher rates of tobacco use and alcohol consumption (1,4,21,24).

Men also tend to have lower rates than women of handwashing, social distancing, wearing masks, and effectively and proactively seeking medical help (1,4,21,25,26). Many men have been socialized to mask their fear, and it is important to consider how hiding fear affects men’s response to COVID-19 (27). It is particularly important to focus on men who respond to threats like COVID-19 with aggression and anger. Research shows that people with this response “tend to downplay risk and are resistant to risk reduction policies,” which is problematic during efforts to promote social distancing and other pandemic restrictions (27). These socially constructed behaviors reduce the perception of susceptibility and severity, which then translates into a decrease in the practice of preventive measures, such as handwashing, and protests against pandemic-related restrictions.

Other factors may intersect with sex and gender, such as age and geography (28). For example, a US study of associations between perceived risk and worry with age and gender found that although older men perceived their risks of COVID-19 to be higher than those of younger men, older men made the fewest behavior changes across age and gender groups (29). Another study highlighted the importance of considering place or geography. In a comparison of counties where populations were predominantly Black or predominantly White, the SARS‐CoV‐2 infection rate was 3 times higher and the death rate was 6 times higher in counties where the population was predominantly Black (30). In urban areas with high percentages of Black residents with low socioeconomic status, some problematic narratives have emerged that blame the men and women who live in these areas for their high rates of COVID-19 rather than the policies or structures that create these conditions (31).

In addition to these psychological and behavioral factors, differences in occupational risk exist between men and women. In the United States, a larger number of women than men are deemed essential workers primarily because of the large share of women employed as social workers and in health care (32). Nevertheless, the low-skilled or low-paid occupations that are considered essential workers (eg, food processing, transportation, delivery, warehousing, construction, manufacturing), where men outnumber women, seem to be associated with a greater risk of mortality (32).

In summary, a range of biological, psychological, and behavioral factors can explain why men have higher rates of COVID-19–associated morbidity and mortality than women. Although it is critical to identify the factors associated with increased risk for men of COVID-19 mortality, it is equally important to determine how to reduce the risk of men dying of COVID-19 (1,4). The factors that exacerbate men’s risk also are intertwined with race, ethnicity, geography, and other proxies for factors that are markers of marginalization and social inequality (4,14). In the remainder of this commentary, we will discuss selected examples of what can be done, and is being done, to reduce men’s risk of COVID-19–associated mortality (Table).

**Table:** Biopsychosocial Determinants and Associated Practice, Policy, and Clinical or Biomedical Intervention Strategies for Reducing Disproportionate COVID-19–Related Morbidity and Mortality Among Men

Determinants (Risk Factors)	Type of Strategy	Strategies (Varying Levels)
**Clinical or Biomedical**		
Comorbidities such as hypertension, cardiovascular disease, chronic kidney disease, diabetes, and chronic obstructive pulmonary disease	Practice	Educate men with comorbidities during routine visits, emergency encounters, and follow-up telephone calls about their susceptibility to COVID-19 and about when to obtain urgent care rather than stay at home for fear of contracting the virus. Reassure patients that new symptoms of myocardial infarction and stroke still need to urgently be addressed.
	Policy	Increase investment in primary prevention of chronic diseases.
Use ACEIs or ARBs	Clinical or biomedical	Physicians and medical researchers should consider consequences of withholding ACEIs or ARBs for men with hypertension. Clinicians should actively assess risks and optimize cardiovascular health.
Sex-dependent immune response and the presence of disease susceptibility genes	Clinical or biomedical	Design clinical trials and population health databases; consider sex as a biological variable that might affect drug efficacy, treatment options, and adverse outcomes. Consider immunologic sex difference in mitigation of disease and clinical trials that consistently investigate sex differences.
ACE2 and TMPRSS2	Clinical or biomedical	Unravel which cellular factors are used by SARS-CoV-2; review for insights into viral transmission; and reveal therapeutic targets for vaccines and medical therapy.
**Behavioral**		
Men who are at increased risk because of cardiometabolic or other preexisting risk factors or are at increased risk because they use tobacco, alcohol, or other drugs	Practice	Focus on helping men who have underlying conditions that increase their risk of COVID-19 mortality to change behaviors that could make it more difficult for their bodies to fight COVID-19–related conditions. Promote American Heart Association’s Life’s Simple 7, including smoking cessation, maintaining a healthy weight, adequate physical activity and balanced healthy diet and target values for cholesterol, blood pressure, and blood glucose
Men who perceive reduced susceptibility and severity of disease and engage in higher-risk behaviors	Policy	Pass risk-reduction policies.
	Practice	Encourage health professionals to educate men on how to reduce viral transmission. Engage men’s partners, families, and trusted loved ones about men’s unique biological or psychosocial risks.
	Clinical or biomedical	Develop and institute COVID-19–specific clinical and operational guidelines in specialties; these include patient education information on occupational risk mitigations, recognizing signs and symptoms of COVID-19 infection, hand hygiene, surface decontamination, and protecting family members.
Men tend to delay seeking clinical care for COVID-19 symptoms	Practice	Eliminate barriers associated with underutilization of health services and improving health literacy. Engage men’s partners and families to support and encourage symptomatic men to seek care. Engage community health workers to provide direct outreach to men with comorbidities to provide culturally and linguistically appropriate preventive care.
	Policy	Increase access to community-wide testing; eliminate costs of testing and other barriers. Collect data related to COVID-19, including data on testing, hospitalizations, intensive care unit admissions, and fatalities, disaggregated by race, ethnicity, sex, and gender at the local and national level to help distribution of resources.

Abbreviations: ACE2, angiotensin-converting enzyme 2; ACEI, angiotensin-converting enzyme inhibitor; ARB, angiotensin receptor blocker; COVID-19, coronavirus disease 2019; SARS-CoV-2, severe acute respiratory syndrome coronavirus 2; TMPRSS2, transmembrane protease, serine 2.

## Intervention Strategies to Reduce Men’s COVID-19 Mortality Risk

To reduce virus transmission and increase screening for the virus and thereby reduce men’s risk of COVID-19 mortality, we propose 5 strategies: 1) health education, community engagement, and public health outreach; 2) health promotion and preventive care; 3) sex-disaggregated data in clinical practice and policy; 4) rehabilitation and health care delivery infrastructure; and 5) health policy and legislative interventions (Figure).

**Figure:**
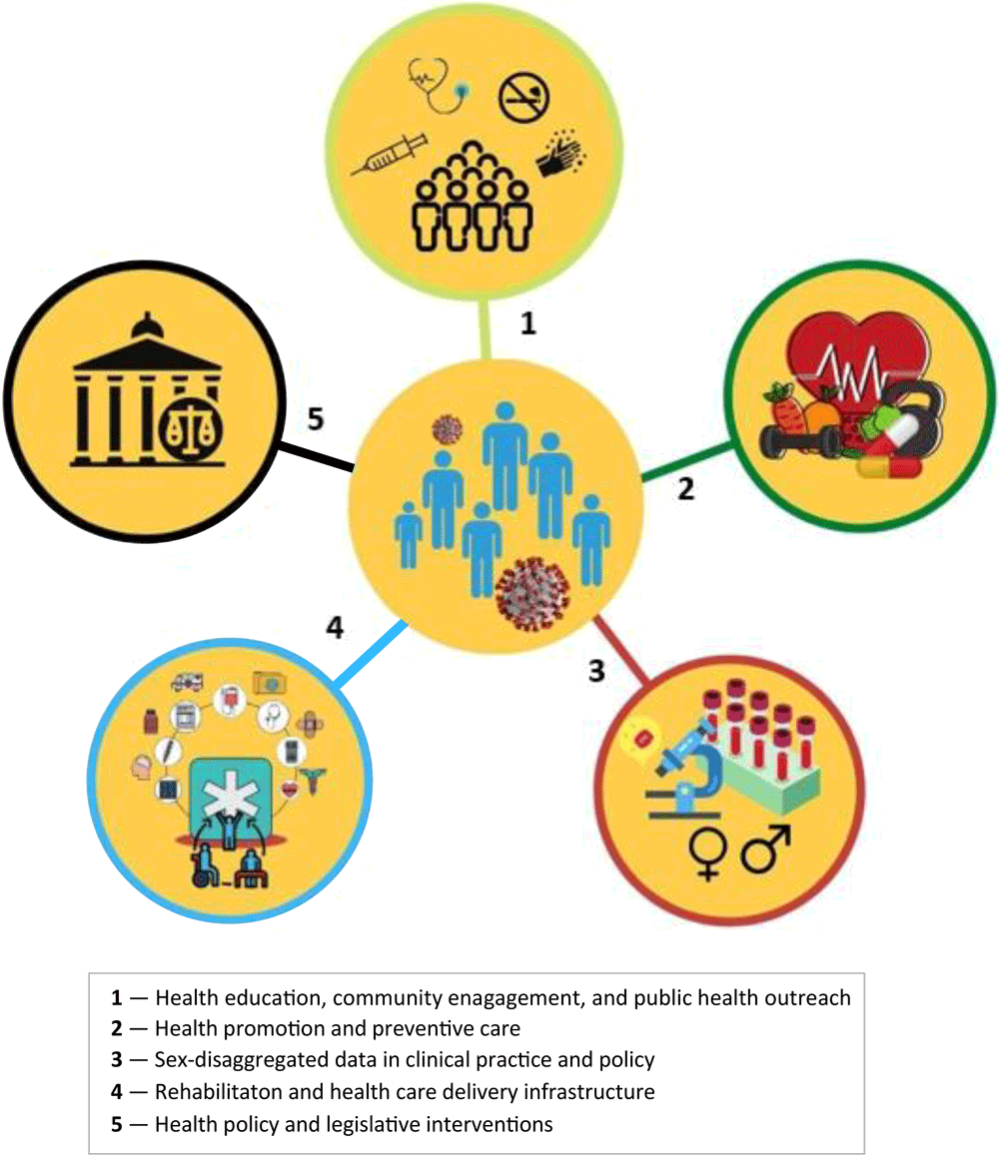
Intervention strategies to reduce men’s COVID-19 mortality risk.

### Health education, community engagement, and public health outreach

Educational efforts to increase compliance with public health recommendations may be more effective in changing the behavior of men if these efforts incorporate some of the principles from health communications research that consider how health behavior is gendered (33,34). Building on research examining psychosocial barriers to men’s health-promoting behaviors (34,35), we note the importance of exploring how men’s priorities, values, and goals are affecting their choices to follow or ignore COVID-19–related transmission prevention messages and pay attention to or ignore potential symptoms that may be present in their bodies. Building on principles of the self-determination theory, we suggest that messages to engage men seek ways to motivate them to consciously choose to engage in healthier behaviors, not because of shame, pressure, or coercion but because they are intrinsically motivated to do so (36). For example, some men may be motivated to engage in behaviors to reduce their risk of contracting or potentially transmitting COVID-19 not by focusing on their risk but by focusing on the high rates of morbidity or mortality of their racial or ethnic group, communities, neighborhood, or family. Being motivated by one’s own reasons to follow COVID-19–related transmission prevention messages is critical when men are faced with pressures to go back to work, the desire to spend leisure time with friends and family, and the inconvenience and fatigue of wearing face masks and gloves or maintaining physical distance from others.

Although the health education of men is useful, the health education of men’s partners and their families about men’s health risks is also critical. One US study of communication strategies examined the influence of men’s partners and found that communicating with a man’s loved one, combined with a reminder system implemented by providers, was associated with increases in preventive health care screenings (37). As a result, a federally qualified health center in Baton Rouge, Louisiana, for example, is conducting outreach to men with underlying conditions and their partners to ensure that they are aware of their susceptibility to COVID-19.

Increasing access and eliminating barriers to community-wide testing are additional ways to improve COVID-19 outcomes. Testing or screening use may be influenced by exposure to decision education and the influence of screening-related primary care practice factors (38). Federally qualified health centers offering primary care services are key community institutions that have increased COVID-19 testing — with no out-of-pocket costs to patients in many areas. These kinds of programs allow men to have access to testing without cost barriers that may otherwise deter them from accessing testing. The community-wide testing also offers an opportunity for men to be tested before returning to work as states begin to reopen and more services (barber shops, gyms, restaurants) are offered in communities. These initiatives help to normalize testing and reduce the stigma of getting tested, although they may not reduce the stigma of receiving a positive test result.

### Health promotion and preventive care

Given the rates of cardiometabolic risk factors and underlying or preexisting conditions such as obesity or comorbid chronic diseases (eg, diabetes, heart disease, cancer) among men, a focus on men with underlying conditions that increase their risk of COVID-19 mortality is critical (34,37). Although the greater severity of complications attributable to COVID-19 among men is not well understood, preliminary findings of a higher incidence of mortality attributable to underlying comorbid conditions suggest that clinicians tailor current treatment options with this in mind. A model that examined activations for ST-segment elevation myocardial infarction (STEMI), the time from coronary artery occlusion to coronary blood flow restoration, showed a significant drop of 38% from roughly the year before the outbreak (January 2019) to the first month of it (March 2020) (25). The study, which used data from 9 high-volume cardiac catheterization laboratories, showed that total STEMI activations decreased from more than 180 per month (mean, 23.6 per center) to only 138 activations per month (mean, 15.3 per center) Thus, patients might be staying at home for fear of contracting the virus even though they need urgent care. We need to reassure patients that although routine and elective care might be curtailed by the pandemic, new symptoms of myocardial infarction and stroke still need to be immediately addressed.

For men who are at increased risk because of a history of a chronic condition or disease, clinicians should actively assess risks; optimize antihypertensive and statin therapies where indicated; provide behavioral and pharmacotherapy for tobacco use cessation (cigarettes and vaping); educate on healthy diets rich in vegetables, legumes, grains, fruits and nuts; and make exercise recommendations (39). In addition to providing information, clinicians should encourage men to participate in behavioral interventions that target psychosocial factors (eg, self-efficacy, motivation) that can facilitate lifestyle change and maintenance of behavior changes over time (34). These important interventions should continue during a pandemic through virtual visits and telemedicine platforms. Several professional organizations have made COVID-19–specific clinical and operational guidelines in their specialties; these include patient education information on occupational risk mitigations and recognizing signs and symptoms of COVID-19 infection, hand hygiene and surface decontamination, and protecting family members (40,41).

### Sex-disaggregated data in clinical practice and policy

While designing clinical trials to address COVID-19–related conditions, clinicians and researchers need to consistently consider sex as a biological variable and the behaviors and social stressors associated with gender that might affect drug efficacy, treatment options, and adverse outcomes (3,13). There is a long history of not analyzing and reporting sex differences and underrepresenting women in cardiovascular clinical trials and in the treatment of infectious diseases (10), and COVID-19 is proving no different in many countries (4,15). Results from the randomized, controlled Adaptive COVID-19 Treatment Trial, which tested remdesivir as a therapeutic agent for the treatment of COVID-19, showed a 4-day difference in time to recovery between the treatment group and the control group, but the study did not provide explicit information on sex-based efficacy or adverse reactions (42). An immunologic sex difference may exist in the mitigation of COVID-19, yet 86% of participants enrolled in clinical trials of immunotherapies (eg, tocilizumab) are men (43). Only by investigating sex differences consistently, critically, and reflectively can we fulfill the requirements of scientific rigor, excellence, and maximum impact.

### Rehabilitation and health care delivery infrastructure

Strategies aimed at preventing complications associated with COVID-19 are essential for safe and effective return to personal, professional, and societal obligations. Urgent needs also exist to provide post–acute care rehabilitation services for patients recovering from COVID-19 and to train a new workforce to care for these patients (44). Strong evidence suggests that interventions engaging community health workers improve health outcomes for patients, including men, across multiple chronic conditions. As care extenders, community health workers provide a culturally and linguistically appropriate clinical–community linkage for difficult-to-reach patients, such as men. They can provide direct outreach to men with comorbidities that make them more susceptible to COVID-19 and its complications.

Given the high rates of pre-existing chronic conditions among men (1), the Center for Medicare and Medicaid Services may need to expand access to telehealth services for men to receive care where they are to allow them to remain in isolation and prevent spread of the virus; however, most assisted living and long-term care facilities do not have computer access for residents for this purpose. This patient-centered care delivery model could be a particularly useful strategy to increase access to preventive medicine for men who are from medically underrepresented groups or groups with lower socioeconomic status (45).

### Health policy and legislative interventions

In addition to various practice initiatives to reduce virus transmission and mortality, we must also consider the potential policy efforts to address the COVID-19 epidemic in the United States. Because men are dying of COVID-19 disproportionately, policy makers need to explicitly consider gender but not conflate gender with women (1). To do so, local, state, and national policy makers should ensure that legislation includes language that promotes data collection, disaggregation, and dissemination by race, ethnicity, and sex (1,4,15). Collecting and disseminating data by sex may help to make a vital economic case for considering men’s health explicitly in the COVID-19 pandemic; however, men’s health policy needs to be located in a framework that embraces gender equity and that does not treat men’s health and women’s health as though they are competing interests or priorities (1). Finally, it is essential for policy makers to adopt an equity-based approach that considers the heterogeneity among men (1,12). Men who are marginalized or disadvantaged because of their race, ethnicity, sexual orientation, incarceration, homelessness, or other factor are particularly vulnerable to COVID-19 and policies should explore which groups of men are overrepresented among essential workers, at risk because of preexisting health conditions, or most in need because of other socioeconomic factors.

## Public Health Implications

A biopsychosocial approach takes into account not only the range of factors that determine risk but also the range of places where we might intervene within a population health framework that considers both biomedical and public health points of intervention to reduce mortality from COVID-19. We must ensure that COVID-19 screening, testing, and quarantine of all confirmed and potential cases; contact tracing; financing; and development of vaccines and clinical trials for novel therapeutic targets do not vary by sex or other socially meaningful markers of difference in our society. Moreover, we need to dramatically increase our investment in the prevention and control of chronic diseases such as hypertension, diabetes, cardiovascular diseases, chronic renal disorders, and mental health disorders that may help us to reduce COVID-19 mortality among men. We can seize this moment to reimagine and redesign our health care and public health systems to consider men’s health, which would have significant benefits for our health care institutions, public health system, and economy.
